# Diffuse myocardial fibrosis evaluation using cardiac magnetic resonance T1 mapping: sample size considerations for clinical trials

**DOI:** 10.1186/1532-429X-14-90

**Published:** 2012-12-28

**Authors:** Songtao Liu, Jing Han, Marcelo S Nacif, Jacquin Jones, Nadine Kawel, Peter Kellman, Christopher T Sibley, David A Bluemke

**Affiliations:** 1 Radiology and Imaging Sciences, National Institutes of Health Clinical Center, Bethesda, MD, USA; 2 Molecular Biomedical Imaging Laboratory, National Institute of Biomedical Imaging and Bioengineering, Bethesda, MD, USA; 3 U.S. Food and Drug Administration, Rockville, MD, USA; 4 Laboratory of Cardiac Energetics, National Heart, Lung and Blood Institute, Bethesda, MD, USA

## Abstract

**Background:**

Cardiac magnetic resonance (CMR) T1 mapping has been used to characterize myocardial diffuse fibrosis. The aim of this study is to determine the reproducibility and sample size of CMR fibrosis measurements that would be applicable in clinical trials.

**Methods:**

A modified Look-Locker with inversion recovery (MOLLI) sequence was used to determine myocardial T1 values pre-, and 12 and 25min post-administration of a gadolinium-based contrast agent at 3 Tesla. For 24 healthy subjects (8 men; 29 ± 6 years), two separate scans were obtained a) with a bolus of 0.15mmol/kg of gadopentate dimeglumine and b) 0.1mmol/kg of gadobenate dimeglumine, respectively, with averaged of 51 ± 34 days between two scans. Separately, 25 heart failure subjects (12 men; 63 ± 14 years), were evaluated after a bolus of 0.15mmol/kg of gadopentate dimeglumine. Myocardial partition coefficient (λ) was calculated according to (ΔR1myocardium/ΔR1blood), and ECV was derived from λ by adjusting (1-hematocrit).

**Results:**

Mean ECV and λ were both significantly higher in HF subjects than healthy (ECV: 0.287 ± 0.034 vs. 0.267 ± 0.028, p=0.002; λ: 0.481 ± 0.052 vs. 442 ± 0.037, p < 0.001, respectively). The inter-study ECV and λ variation were about 2.8 times greater than the intra-study ECV and λ variation in healthy subjects (ECV:0.017 vs. 0.006, λ:0.025 vs. 0.009, respectively). The estimated sample size to detect ECV change of 0.038 or λ change of 0.063 (corresponding to ~3% increase of histological myocardial fibrosis) with a power of 80% and an alpha error of 0.05 for heart failure subjects using a two group design was 27 in each group, respectively.

**Conclusion:**

ECV and λ quantification have a low variability across scans, and could be a viable tool for evaluating clinical trial outcome.

## Background

Diffuse myocardial fibrosis (DMF) is a common histological feature of the failing heart and is present in many conditions, ranging from advanced aging to hypertension or hypertrophic cardiomyopathy [1-3]. DMF is thought to be primarily responsible for increased myocardial stiffness and diastolic dysfunction: an increasingly common condition in the elderly [4,5]. Endomyocardial biopsy (EMB) is the standard of reference for quantifying DMF, but is an invasive procedure and prone to sampling error [6,7].

Myocardial composition may be probed noninvasively by measuring the T1 time of the myocardium, termed T1 mapping. DMF results in increased collagen content with expansion of the extracellular space to a greater extent than that of normal myocardium [8,9], resulting in accumulation of gadolinium-based contrast agents (GBCA). This, in turn, lowers the T1 time of the myocardium. Altered myocardial T1 times have been demonstrated in a range of nonischemic cardiomyopathies [10], including chronic aortic regurgitation [11], heart failure [7], aortic stenosis [12], and adult congenital heart disease [13].

Unfortunately, absolute quantification of T1 time is influenced by many factors, including the relaxivity of the GBCAs, the delay time after injection, and renal function (glomerular filtration rates, GFR) [14]. As an alternative, other indices of DMF have been considered, such as extracellular volume fraction (ECV) and partition coefficient (λ) [15-18]. Of note, there is considerably less change in ECV over time at steady state compared to relatively large changes in T1 values as a function of time after GBCA injection [19,20]. In addition, ECV is relatively robust as a function of field strength [21]. Thus, ECV and partition coefficient are likely to be more favorable measures to determine change in DMF as a result of treatment or disease.

Therapeutic agents targeted at reducing DMF have been actively investigated in animal models [22-24]. To date, no human prospective studies with the goal of reducing DMF have been reported. In order to provide utility as a biomarker for longitudinal studies, one must estimate the test, re-test (inter-study) reproducibility of ECV and partition coefficient. Inter-study reproducibility in turn is affected by factors such as measurement error (e.g. due to patient motion or reader variability), variation in MRI scanner performance or pulse sequences and contrast agents. Knowledge of inter-study reproducibility can be used to estimate the sample size needed to demonstrate a statistically significant change in ECV or partition coefficient.

The purpose of this study was to estimate the variability of quantitative T1 measurements and, in particular, of the derived values of ECV and partition coefficient. We then provide sample size estimates to determine the potential of cardiac magnetic resonance (CMR) T1 data to be used as a noninvasive biomarker aimed at identifying reduction of DMF in response to a therapeutic intervention.

## Methods

### Study population

This study was approved by our institutional review board. All study participants provided written informed consent. Twenty-four healthy volunteers (8 men; 29 ± 6 years) without a history of cardiovascular or systemic disease were enrolled. The ECG obtained prior to the CMR exam did not show any abnormality and the physical exam performed by a physician did not reveal any pathologic finding. Normal left ventricular (LV) and right ventricular (RV) volumes and systolic functions were confirmed by CMR. The healthy subjects’ data has been previous published by our group [25]. In addition, twenty-five heart failure (HF) subjects (12 men; mean age ± SD, 63 ± 14 years) with NYHA classification II or greater were enrolled.

### CMR protocol

All CMR exams were performed using a 3-Tesla scanner (Verio, Siemens Medical Systems, Erlangen, Germany) with a 32-channel cardiovascular array coil. T1 quantification was performed with a modified Look-Locker with inversion recovery (MOLLI) sequence [26] acquired during end-expiratory apnea in a mid-ventricular short axis view before and 12, and 25 minutes after GBCA. The MOLLI protocol has two inversion blocks; three images are acquired after the first inversion pulse, followed by a pause of three heart beats, then five images are acquired after a second inversion pulse [20]. Other CMR parameters were: non-segmented, steady state free precession read out in mid-diastole; FOV 290 to 360 mm; readout resolution 192; phase resolution 75% to 85%; slice thickness 8 mm; TR/TE 1.9/1.0ms, minimum inversion time 110ms, inversion time increment 80ms, flip angle 35°; GRAPPA parallel imaging factor 2, no partial Fourier in the phase encode dimension.

GBCA was injected intravenously at 2 ml/sec using a power injector and followed by a 20ml saline bolus administered at the same flow rate. Both healthy and HF subjects underwent CMR examination with 0.15mmol/kg of gadopentate dimeglumine. Healthy volunteers also underwent another CMR examination with the same CMR protocol with 0.1mmol/kg of gadobenate dimeglumine. The mean delay between the two studies was 51 ± 34 days.

Left ventricular volume and function were evaluated with steady state free precession cine imaging in short axis stack and in three long axis views. Late gadolinium enhancement (LGE) was acquired in the same position as cine images using a phase sensitive inversion recovery gradient echo sequence [27] after 15min of GBCA injection. Blood samples were taken 1 to 4 hours prior to the CMR to determine the HCT and creatinine.

### Image analysis

T1 maps were generated by three points pixel-wise curve fitting [28] and stored in Digital Imaging and Communications in Medicine (DICOM) Format. To extract myocardial T1 value, endocardial and epicardial contours were manually traced using QMass MR 7.2 (Medis, Leiden, Netherlands), and the myocardial circumference was divided into segments according to the American Heart Association 17-segment model [29]. Care was taken to exclude epicardial structures and blood from the contours. T1 value of the blood pool was measured by manually drawing a region of interest in the left ventricular cavity excluding papillary muscles. The image quality for all segments was visually rated using a scale in which a score of 3 indicated that image quality was good, with no artifacts; a score of 2, that image quality was satisfactory, with minor artifacts; and a score of 1, that an image was non-evaluable with major artifact, as described by Messroghli [30]. T1 values from segments that were rated as non-evaluable were excluded from analysis. ECV and λ values were calculated according to the following formulae [15]:

(1) $$\mathrm{\Delta R}1_{\text{myo}}=1/T1_{\text{myo}-\text{post}}-1/T1_{\text{myo}-\text{pre}}$$

(2) $$\mathrm{\Delta R}1_{\text{blood}}={1/T1}_{\text{blood}-\text{post}}-1/T1_{\text{blood}-\text{pre}}$$

(3) $$\lambda =\mathrm{\Delta R}1_{\text{myo}}/\text{ΔR}1_{\text{blood}}$$

(4) $$\mathrm{ECV}=\lambda \times \left(100-\text{HCT}\right)$$

Where ECV, λ, and HCT are given as percentages.

### Statistical analysis

Statistical analyses were performed using SAS 9.1 (Cary, North Carolina, USA) and MedCalc 12.2 (MedCalc Software, Mariakerke, Belgium). Sample size estimation was performed using PASS 2008 (Kaysville, Utah, USA). For comparison of the means between groups, one-way analysis of variance with post-hoc comparison was performed. Data are presented as mean ± standard deviation. Statistical significance was defined as *P* < 0.05.

The intra-study and inter-study reproducibility were assessed by calculating the difference and standard deviation between results. The coefficient of variability was calculated as the standard deviation of the difference divided by the mean of the parameter under consideration. Intra-study reproducibility compares the difference of ECV and partition coefficient at the 12-minute and 25-minute time points of the *same* study session: this is the best case scenario for testing ECV and partition coefficient reproducibility. Inter-study reproducibility, which compares ECV and partition coefficient results of two *different* study sessions, is the standard test-retest reliability. The inter-study reproducibility – the standard deviation of the mean difference – is the key factor for determining the ability of a technique to perform longitudinal examinations to detect a change. High reproducibility (low inter-study standard deviation) leads to greater reliability of observed changes in a parameter and a smaller sample size in clinical trials.

The sample size required by ECV or λ to show a clinical change with a power of 80% and an α error of 0.05 were calculated using the following formula:

(5) $$n=f\left(a,P\right)•\sigma^{2}•2/\delta^{2}$$

Where n is the sample size needed, α is the significant level, *P* is the study power required, and f is the value of the factor for different values of α and P, with σ as the inter-study standard deviation and δ as the desired difference to be detected [31,32].

## Results

Study subject characteristics are given in Table 1. CMR was well tolerated by all subjects in the study. Both ECV and λ were significantly higher in the heart failure group compared to the healthy group (ECV: 0.287 ± 0.034 vs. 0.267 ± 0.028, p = 0.002; λ: 0.481 ± 0.052 vs.442 ± 0.037, p < 0.001). For the healthy group, there was no statistical difference between 12 minute and 25 minute ECV and λ (ECV: 0.264 ± 0.028 vs. 0.271 ± 0.028, p = NS; λ:0.436 ± 0.038 vs. 0.447 ± 0.037, p = NS). In addition, there was no significant difference for these parameters between gadopentetate dimeglumine and gadobenate dimeglumine (ECV: 0.271 ± 0.027 vs. 0.264 ± 0.029, p = NS; λ: 0.449 ± 0.039 vs. 0.435 ± 0.035, p=NS). Similarly, there was no statistical difference between 12 minute and 25 minute ECV and λ (ECV, 0.282 ± 0.033 vs. 0.289 ± 0.034, p = NS; λ: 0.475 ± 0.053 vs. 0.487 ± 0.051, p = NS) in the heart failure group. These results confirm the stability of ECV over moderate time intervals, and suggest a similar biodistribution of the two contrast agents. Of note, the image quality of T1 maps was significantly better in healthy group (2.8 ± 0.2 for healthy, 2.6 ± 0.4 for heart failure, p < 0.001).

**Table 1:** Participant characteristics

**Demographics**	**Normal subjects (n=24)**	**Heart failure subjects (n=25)**
Age	28.6 ± 5.9	62.7 ± 14.3
Male	8 (33.3)	12 (48.0)
Hematocrit (%)	39.7 ± 3.8	40.5 ± 3.1
Serum creatinine (mg/dL)	0.75 ± 0.15	0.91 ± 0.28
eGFR (ml/min)	115.5 ± 21.5	82.3 ± 18.5
**Medical history**		
Diabetes Mellitus(%)	0 (0)	2 (8.0)
Smoking	3 (12.5)	1 (4.0)
Hypertension	0 (0)	14 (56.0)
Hyperlipidemia	0 (0)	8 (40.0)
**LV function by CMR**		
EDV (ml)	147.6 ± 31.8	214.8 ± 116.5
ESV (ml)	56.5 ± 14.9	133.9 ± 104.5
EF (%)	61.9 ± 4.0	42.1 ± 18.7
Mass (g)	111.0 ± 36.6	203.0 ± 110.6
Stroke volume (ml)	91.1 ± 19.4	81.0 ± 43.7

Note: Mean and standard deviation or number and percentage as appropriate. LV, left ventricular; EDV, end-diastolic volume; ESV, end-systolic volume; EF, ejection fraction.

### Repeat measures of ECV and λ, intra-study assessment

The intra-study data of ECV and λ for both normal and HF groups are shown in Table 2. As expected, the correlation between the 12 minute and 25 minute of ECV and λ in the same study session was better in healthy subjects (0.98, 0.97) compared with that of the heart failure patients (0.88, 0.86). ECV has smaller Bland-Altman limits of agreement and intra-study standard deviation compared with partition coefficient (Table 2). The intra-study variability of both ECV and λ was larger in the heart failure group compared to that of the healthy group.

**Table 2:** Intra-study reproducibility data in healthy and heart failure groups

	**Healthy subjects**		**Heart failure subjects**	
	λ	**ECV**	λ	**ECV**
Mean ± SD	0.442 ± 0.037	0.267 ± 0.028	0.481 ± 0.052	0.286 ± 0.034
Min: Max	0.367 : 0.530	0.202 : 0.325	0.368 : 0.634	0.240 : 0.398
Mean Diff ± SD	0.012 ± 0.009	0.007 ± 0.006	0.012 ± 0.028	0.007 ± 0.017
Corr Coef	0.97	0.98	0.86	0.88
CV	0.020	0.022	0.058	0.059
BA limit	-0.03 : 0.006	-0.018 : 0.004	-0.068 : 0.043	-0.041 : 0.026

λ: partition coefficient; ECV: extracellular volume fraction; Mean Diff, mean difference; Corr Coef, correlation coefficient; CV, coefficient of variability; BA limit, Bland-Altman limits of agreement.

### Inter-study difference and sample size estimation

The inter-study data of ECV and λ of the healthy group are shown in Table 3. Compared with the same intra-study data parameters, the correlation coefficients were lower for data acquired at a different study session. As expected, the CV and Bland-Altman limits of agreement of inter-study were also greater compared with that of the intra-study. Pre-contrast myocardial T1 exhibits high agreement between two study sessions.

**Table 3:** Inter-study reproducibility data in healthy subjects

	**Pre-contrast Myocardium T1 (ms)**	**λ**	**ECV**
Mean ± SD	1159.0 ± 39.2	0.442 ± 0.037	0.267 ± 0.028
Mean Diff ± SD	-9.4 ± 29.2	-0.016 ± 0.025	-0.006 ± 0.017
Corr Coef	0.75	0.78	0.82
CV	0.025	0.057	0.064
BA limit	-47.8 : 66.6	-0.033 : 0.066	-0.027 : 0.040

λ : partition coefficient; ECV: extracellular volume fraction; Mean Diff, mean difference; Corr Coef, correlation coefficient; CV, coefficient of variability; BA limit, Bland-Altman limits of agreement.

In healthy subjects, the inter-study SD of ECV and λ were about 2.8-fold greater than the intra-study (ECV: 0.017 vs. 0.006; λ 0.025 vs. 0.009). In heart failure subjects, the intra-study of ECV and λ were 0.017 and 0.028, respectively. The sample size needed for the heart failure group was estimated for three different cases:

Case 1 Inter-study SD of ECV and λ estimated at 2.8 times greater than the intra-study SD (SD1 and N1 in Table 4),

(6) $$\begin{array}{c} \text{ECV}\_SD_{\;\text{int}\text{er}-\text{study}}=\text{ECV}\_SD_{\;\text{int}\text{ra}-\text{study}}\times 2.8=0.017\times 2.8\\ \;=0.048\\ \;\lambda \_SD_{\;\text{int}\text{er}-\text{study}}=\lambda \_SD_{\;\text{int}\text{ra}-\text{study}}\times 2.8=0.028\times 2.8\\ \;=0.078 \end{array}$$

**Table 4:** Estimated sample size in heart failure group to detect the change of ECV and λ with a power of 80%

**Clinical change**	**Caset 1**		**Case 2**		**Case 3**	
	**SDD**_**1**_	**N**_**1**_	**SDD**_**2**_	**N**_**2**_	**SDD**_**3**_	**N**_**3**_
λ (0.063)	0.078	26	0.117	56	0.156	98
ECV (0.038)	0.048	27	0.072	58	0.096	102

Sample size need to detect a clinical meaning change of ECV and λ with 80% of power and an alpha error of 0.05. Sample size is derived from the inter-study SDD as described by Altman [33] and Marchin [32]. Note that for studies comparing active vs. placebo, these sample size numbers need to be doubled. Case 1: the inter-study SDD_1_ in HF group was estimated 2.8 fold greater than the intra-study SDD; Case 2, the inter-study SDD_2_ was estimated 1.5 times more than SDD_1_; Case3, the inter-study SDD_3_ was estimated 2 times more than SDD_1_.

Case 2 50% more variation than Case 1: Inter-study SD of ECV and λ estimated at 4.2 times greater than the intra-study SD (SD2 and N2 in Table 4),

(7) $$\begin{array}{l} \mathrm{ECV}\_SD_{\;\text{int}\text{er}-\text{study}}=\text{ECV}\_SD_{\;\text{int}\text{ra}-\text{study}}\times 4.2=0.017\times 4.2\\ \;=0.072\\ \;\lambda \_SD_{\;\text{int}\text{er}-\text{study}}=\lambda \_SD_{\;\text{int}\text{ra}-\text{study}}\times 4.2=0.028\times 4.2\\ \;=0.117 \end{array}$$

Case 3 100% more variation than Case 1: Inter-study SD of ECV and λ estimated at 5.6 times greater than the intra-study SD (SD3 and N3 in Table 4),

(8) $$\begin{array}{l} \text{ECV}\_SD_{\;\text{int}\text{er}-\text{study}}=\text{ECV}\_SD_{\;\text{int}\text{ra}-\text{study}}\times 5.6=0.017\times 5.6\\ \;=0.096\\ \;\lambda \_SD_{\;\text{int}\text{er}-\text{study}}=\lambda \_SD_{\;\text{int}\text{ra}-\text{study}}\times 5.6=0.028\times 5.6\\ \;=0.156 \end{array}$$

### Sample size estimation

In patients without LGE, the median percent histological fibrosis was 6.5% with inter-quartile range of 3.0 – 9.0% at endomyocardial biopsy [34]. Therefore, a 3% increase of histological fibrosis represents 25% more myocardial fibrosis over baseline would be clinically meaningful. The correlation coefficient between ECV quantification and histological fibrosis was 0.69 in a rat hypertension model [35]; and 0.89 in aortic stenosis and hypertrophic cardiomyopathy patients [12]. Take the average ECV correlation coefficient and the proposed 3% increase of histological fibrosis translates into a clinically meaningfully ECV change of 0.038 or lambda change of 0.063, assuming a hematocrit of 0.4.

**Figure 1:**
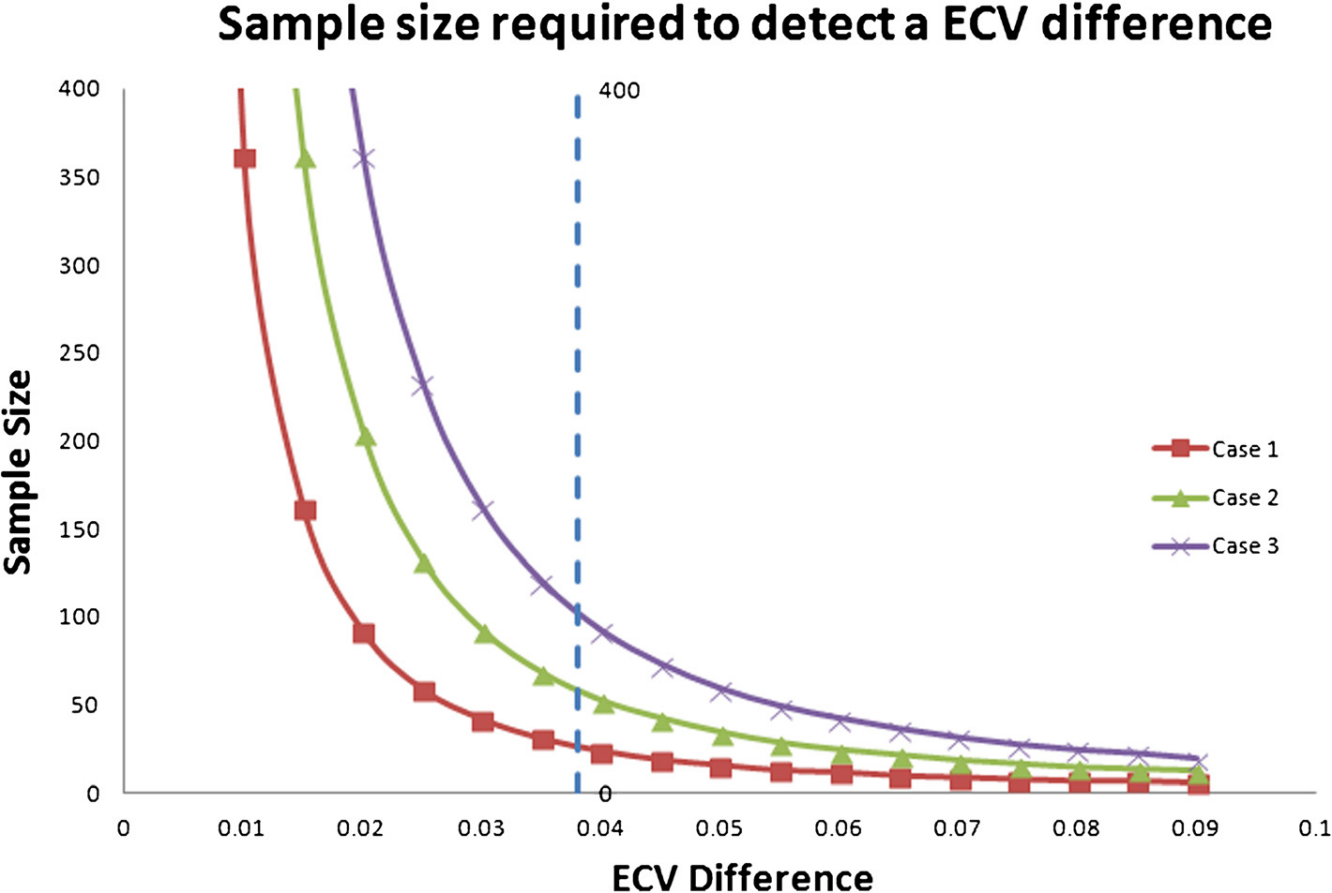
**Sample size required in each group to detect a certain ECV difference with a two group design of 80% power and an alpha error of 0.05.** The X axis values corresponding to the ECV difference need to be detected like the first column in Table 4. The three curves corresponding to case 1, 2 and 3 of Table 4. The smaller ECV difference and higher inter-study SD, the larger the sample size needed. The dashed line corresponding to the sample size needed to detect a 0.038 ECV difference for the three cases as showed in Table 4.

**Figure 2:**
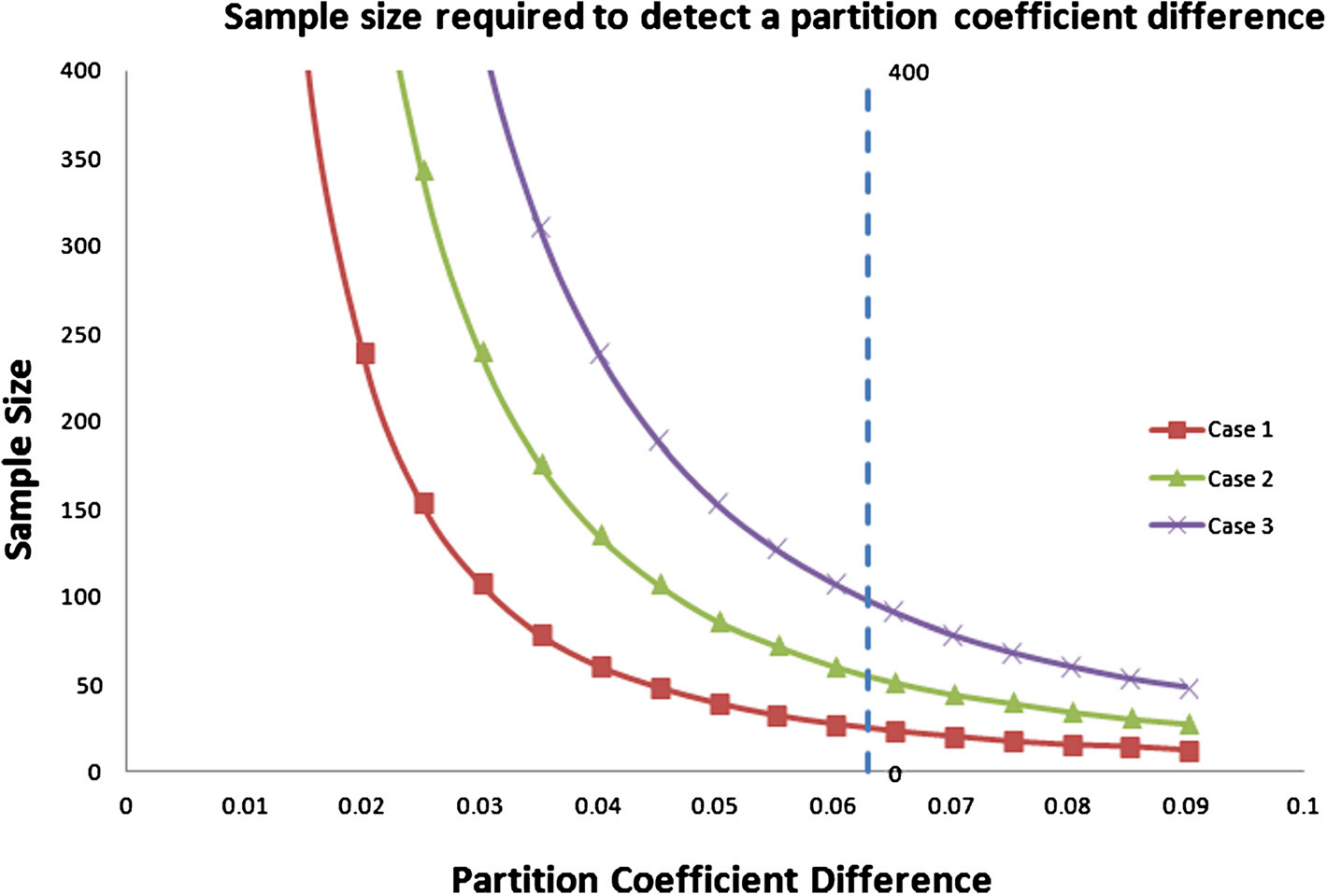
**Sample size required in each group to detect a certain partition coefficient difference with a two group design of 80% power and an alpha error of 0.05.** The X axis values corresponding to the partition coefficient difference need to be detected like the first column in Table 4. The three curves corresponding to case 1, 2 and 3 of Table 4. The smaller partition coefficient difference and higher inter-study SD, the larger the sample size needed. The dashed line corresponding to the sample size needed to detect a 0.063 partition coefficient difference for the three cases as showed in Table 4.

For Case 1, 27 patients would be needed to detect a 0.038 change in ECV or 0.063 change in λ with 80% of power. For a “worst case” scenario with more variability as in Case 3 (e.g., a multi-center trial), 100 patients would be needed to detect a 0.038 change in ECV or 0.063 change in λ with 80% of power. For studies comparing active treatment vs. placebo, these sample size numbers need to be doubled. Figure 1 and Figure 2 show the sample sizes required for detection of a certain ECV or λ difference with a power of 80% and an alpha error of 0.05 under different inter-study standard deviations.

## Discussion

DMF is a common endpoint associated with a wide range of cardiomyopathies. Preclinical studies have shown a reduction in DMF in response to angiotensin converting enzyme inhibitors [24,36,37] and N-acetylcysteine [22,23]. For a similar human clinical trial, a paired study design offers more power to assess treatment response than an unpaired design. In this analysis, we provide estimates that are useful for such a paired study design, allowing the following conclusions: a) sample size needed to detect a meaningful clinical change are similar for ECV and partition coefficient; b) sample size estimates become highly sensitive to the inter-study reproducibility for a target change in ECV of less than 0.04-0.05; and c) sample sizes of 50-100 subjects in each study arm are likely to be necessary to detect changes of 0.03-0.05 in ECV for inter-study standard differences on the order of 0.05. Note that these sample size estimates would be equally applicable to a scenario that sought to halt progression of DMF, under the assumption that DMF would otherwise show a defined rate of increase over time.

CMR using LGE technique has been the standard of reference for detecting focal myocardial replacement fibrosis or scarring fibrosis in conditions such as myocardial infarction and hypertrophic cardiomyopathy [38,39]. LGE relies on the differences in signal intensity between scarred and adjacent normal myocardium to generate image contrast [27,40]. In an animal model of hypertension-induced DMF, LGE failed to detect any hyper-enhancement while histology analysis revealed an average of 9.9% collagen volume fraction [35]. Similarly, in cardiomyopathy patients, endomyocardial biopsy revealed the presence up to 20% diffuse myocardial fibrosis in patients *without* evidence of LGE [10]. Therefore, the detection of subtle DMF poses a significant challenge to LGE.

Extracellular volume fraction by CMR is a promising tool for visualization and quantification of local and diffuse myocardial abnormalities [15,16,41]. An animal study has demonstrated that elevated ECV was associated with increased collagen deposition [35]. Several human studies have been published using ECV as a surrogate biomarker for DMF [12,13,17,18]. The reproducibility of a technique determines the sample size required to demonstrate a clinical change [42], which is a major cost in clinical trials. Messroghli reported the reproducibility data of myocardial T1 in a group of healthy volunteers [30], but there is a lack of data with regard to the reproducibility of ECV and partition coefficient.

In this study, there is good intra-study agreement between 12 minute and 25 minute ECV and partition coefficient in healthy volunteers, and this compares favorably with previous reports that ECV and partition coefficient are relatively stable after reaching the dynamic equilibrium between myocardium and blood pool [19,43]. The intra-study variability of ECV and λ is higher in heart failure subjects. The primary reason for this was reduced image quality for heart failure subjects. Such patients have reduced capacity for breath-holding, resulting in motion artifacts. The MOLLI protocol used in this study requires a 11-heart-beat breath-hold, 5 heart beats shorter than the classic 17 heart beats MOLLI [44]. An even faster MOLLI protocol, like shMOLLI with 9 heart beats might be helpful in this regard [45]. Xue *et al*. [46] demonstrated a motion correction algorithm using image registration with synthetic image estimation to suppress the motion-induced artifacts in T1 maps. Robust motion correction was achieved by registering synthetic images to the corresponding MOLLI frames, and this method has been incorporated into the inline T1 mapping calculation of some scanners. In the future, a free-breathing T1 acquisition with motion correction would be ideal for the heart failure patients.

High reproducibility (low inter-study standard deviation) leads to greater reliability of observed changes in a parameter. This also results in cost-efficiency, as smaller sample size is required in clinical trials. Our sample size calculation demonstrates that a reasonable sample size is needed to detect a clinically meaningful change in ECV and partition coefficient.

Previously, CMR has successfully shown *group* differences in parameters such as T1 time or ECV between normal versus diseased study subjects [7,15]. In this study, we also demonstrated statistically significant *group* differences in ECV using a relatively small sample size (24 normal subjects versus 25 HF subjects). However, the mean ECV value of the HF subjects (0.286) was within the observed range of values in normal subjects, previously reported to be 0.24-0.27 [13,15,19,20]. Using a cut-value for normal ECV of 0.267, the sensitivity to detect abnormal ECV in HF subjects was only 38%. Thus, ECV is less likely to be useful as single cut-off value to identify abnormal versus normal subjects. However, *change* in ECV *within an individual* may be a more promising approach to assess, for example, a therapeutic response.

There are several limitations to this study. First, we estimated inter-study standardized differences for the heart failure patients using the healthy subjects as a reference group. Repeat gadolinium-enhanced MRI scans over a short interval was not performed due to below normal renal function in the HF group. Our estimates nevertheless appear to be of the correct magnitude. We experimentally detected a statistical significance in ECV with a total sample size of 49 in the study (24 in healthy group and 25 in heart failure group), similar to the 54 total sample size we estimated (27 subjects in each arm with 80% of power and an alpha error of 0.05). In addition, this is a single-center study. All scans were preformed on a single scanner with good adherence to the study protocol. For multi-center studies involving multiple scanners, a higher degree of variation is expected because of the difference of sequences, imagers, coil systems, and field strengths [47]. The inter-study reproducibility is related to sample size by a square function, therefore a much larger sample size is needed to compensate the increased variation in a multi-center study to detect ECV or partition coefficient change (Figure 1 and Figure 2).

## Conclusion

In conclusion, ECV and partition coefficient have a relatively low variability for repeat scans, and could be a viable tool for evaluating clinical trial outcome. Sample size estimation showed that a study with 27 participants in each group could detect a 0.038 change in ECV or 0.063 change in partition coefficient with 80% of power, which corresponding to about 3% increase in histological collagen tissue.

## Competing interests

The authors declare that they have no competing interests.

## Authors’ contributions

All authors read and critically edited the initial manuscript, added intellectual content, and approved the final version. DAB and SL designed, coordinated and conducted the study; JJ recruited subjects; SL, NK and MSN acquired images; SL, NK and MSN analyzed images; JH conducted the statistical analyses. PK assisted with pulse sequence optimization and added critical manuscript content. All authors read and approved the final manuscript.

## Funding sources

Funded by the National Institutes of Health (NIH) intramural program.
